# THE PSYCHOSOCIAL IMPACTS OF AGEISM: FINDINGS FROM SURVEYS IN JAPAN

**DOI:** 10.1093/geroni/igae098.1018

**Published:** 2024-12-31

**Authors:** Masumi Takeuchi

**Affiliations:** Tokyo Metropolitan Institute for Geriatrics and Gerontology, Suginami-ku, Tokyo, Japan

## Abstract

Ageism is a detrimental bias directed towards older people, and understanding its existence and the associated issues is crucial for eradicating ageism. Palmore (1985) posited that in a Confucian society like Japan, older people are respected and ageism is not prevalent. However, subsequent studies have shown that negative prejudices against older people also persist in Japan. This presentation aims to provide an overview of the current state of ageism in Japan and examine its psychosocial impact on older adults. Firstly, I will present the findings of a 2021 survey conducted among 800 individuals aged 60-74, revealing that 75% of respondents reported experiencing some form of age-based discrimination. Employed women encountered age discrimination more frequently than their non-employed counterparts, as well as men. This result is consistent with previous research indicating that older women face a double jeopardy of ageism and sexism in the workplace (Palmore, 1997). Secondly, the data indicates that those who encountered age discrimination exhibited more negative attitudes towards aging and reported poorer subjective well-being. Thirdly, a longitudinal analysis of the “National Survey of the Japanese Elderly,” conducted by the Tokyo Metropolitan Institute for Geriatrics and Gerontology in 1987, 1993, and 2002, among individuals aged 60 and older residing in Japan, demonstrated that older adults with negative attitudes towards aging experienced worse health outcomes and reduced social participation later in life. These findings underscore the pressing issue of ageism in Japan and emphasize the necessity for social initiatives to combat ageism in the country.

